# Back to Work, Running on Empty? How Recovery Needs and Perceived Organizational Support Shape Employees’ Vigor Upon Return to Work

**DOI:** 10.3390/bs15070889

**Published:** 2025-06-30

**Authors:** Yiting Wang, Keni Song, Ming Guo, Long Ye

**Affiliations:** School of Economics and Management, Beijing Jiaotong University, Beijing 100044, China; 21113081@bjtu.edu.cn (K.S.); gming@bjtu.edu.cn (M.G.); yelong@bjtu.edu.cn (L.Y.)

**Keywords:** work-related irritation, need for recovery, workplace vigor, boundary strength at home, perceived organizational support

## Abstract

Returning to work after extended holidays poses significant challenges to employees’ psychological adjustment, yet this phenomenon remains underexplored in organizational research. Drawing on the Conservation of Resources (COR) theory, this study develops and tests a moderated mediation model to examine how pre-holiday work-related irritation influences post-holiday workplace vigor through heightened need for recovery, and how perceived organizational support buffers this process. Data were collected through a four-wave time-lagged design surrounding the Chinese Spring Festival, with a final sample of 349 employees across diverse industries. Results show that pre-holiday emotional strain increases employees’ recovery needs, which in turn undermines their workplace vigor. Moreover, boundary strength at home and perceived organizational support buffer the indirect negative pathway, highlighting the critical roles of both personal and organizational resources in the recovery process. By shifting attention from burnout to positive energy states such as vigor, this study advances theoretical understanding of post-holiday adjustment dynamics and offers practical insights for organizations seeking to foster employee resilience and sustained engagement after structured breaks.

## 1. Introduction

Following extended holidays, many employees experience emotional letdown and psychological discomfort when returning to routine responsibilities. This phenomenon is widely recognized as holiday blues syndrome, a transient stress reaction triggered by the contrast between festive relief and everyday obligations (Baier, 1988). Employees often report feeling unprepared or disconnected when returning to high-performance work settings after time away, especially when the transition is abrupt. Such disruptions not only affect individual short-term productivity but may also have a cumulative impact on team performance, client satisfaction, and broader organizational operations. Despite its widespread occurrence, holiday blues syndrome remains an underexplored area in organizational behavior and human resource management literature.

In many contemporary work environments, particularly those marked by high-intensity demands and limited downtime, employees are expected to resume full capacity immediately after holidays. However, shifting from a restorative, low-pressure context to one of sustained cognitive and emotional demand is rarely seamless. When recovery time proves insufficient to meet renewed expectations, individuals may experience resource depletion, reduced focus, and motivational decline (Kühnel et al., 2017). Left unaddressed, such strain can compromise performance and well-being (van Woerkom et al., 2016). Gaining insight into how employees navigate this vulnerable transition is therefore critical for fostering long-term engagement and organizational sustainability.

Existing studies on occupational stress and recovery have primarily focused on chronic job demands, long-term exhaustion, and gradual burnout processes (Gluschkoff et al., 2016; Eklöf et al., 2022; Hogan et al., 2023). Although this research has yielded important insights, much less attention has been given to short-term resource withdrawal and the challenges employees face in restoring and mobilizing energy following structured breaks such as national holidays or collective leave periods. This gap is particularly critical in East Asian or collectivist cultures (e.g., China), where extended holidays like the Spring Festival are deeply embedded in both social norms and temporal work rhythms. Moreover, the majority of recovery research emphasizes negative affective outcomes, especially emotional exhaustion (Donahue et al., 2012; Sonnentag et al., 2010), while overlooking positive work states such as vigor, which reflect employees’ capacity to invest effort, persistence, and resilience at work.

To address these limitations, this study draws upon the Conservation of Resources (COR) theory (Hobfoll, 1989, 2001) to examine the psychological processes employees undergo as they return to work after extended holidays. COR theory suggests that stress arises when individuals perceive a loss or insufficiency of key personal resources, such as energy or emotional stability, which are essential for work re-engagement (Hobfoll & Freedy, 2017). The return to work after holidays can challenge employees’ ability to mobilize these resources, particularly when recovery time is inadequate. This may result in emotional strain and lower vigor. In this context, perceived organizational support serves as a socio-emotional recovery resource, buffering against further depletion and facilitating smoother adjustment (Halbesleben et al., 2014).

Building on these frameworks, we propose and empirically test a moderated mediation model to better understand the drivers of post-holiday work engagement. We suggest that pre-holiday work-related irritation predicts higher recovery needs after the break and subsequently reduces post-holiday workplace vigor. Furthermore, we examine how this indirect pathway is shaped by two distinct but complementary moderators: (1) boundary strength at home (BSH), reflecting the individual’s capacity to separate work from personal time, and (2) perceived organizational support (POS), capturing the extent to which employees believe their organization values their well-being. Together, these factors reflect both personal boundary strategies and institutional recovery resources.

This study makes several theoretical and practical contributions. First, it is among the few studies to systematically investigate short-term psychological adaptation during the post-holiday period, expanding the temporal boundaries of recovery research beyond daily work–rest cycles. Second, by conceptualizing vigor as the key outcome, rather than focusing solely on strain or burnout, the study offers a positive psychology perspective that aligns with current trends in employee well-being research. Third, incorporating both personal strategies and organizational factors as moderators provides a more nuanced understanding of how these forces jointly influence recovery dynamics. Lastly, the findings have clear managerial implications, offering actionable insights for organizations seeking to optimize post-holiday reintegration, reduce adjustment costs, and sustain employee vitality in high-demand environments.

## 2. Theoretical Framework and Hypothesis Development

### 2.1. COR Theory and Holiday Blues Syndrome

COR theory provides a valuable framework for understanding how individuals respond to resource depletion during transitions from holidays to workplace demands. Although extended holidays are often assumed to replenish such resources, employees returning to work may immediately face performance pressure, cognitive overload, and heightened task demands, all of which collectively tax their remaining capacity. This mismatch may trigger holiday blues syndrome (Baier, 1988), a transient affective condition marked by low mood and diminished work motivation. Empirical research has shown that resource threats following non-work periods can impair psychological re-engagement and increase vulnerability to burnout (Grotto et al., 2022). In this context, post-holiday strain reflects not only incomplete recovery but also the renewed imposition of demands that exceed employees’ available coping resources.

To further unpack the psychological mechanisms underlying this post-holiday strain, we focus on work-related irritation (WRI) as a proximal emotional response to resource misalignment. WRI refers to employees’ emotional reactions, such as restlessness, frustration, or mental agitation, elicited by renewed work-related demands (Mohr et al., 2006). Especially salient after extended breaks, WRI may be intensified by perceived unpreparedness, sudden exposure to information overload, or an immediate surge in task complexity. It reflects an individual’s psychological difficulty in mobilizing sufficient internal resources to meet renewed external demands. Empirical evidence shows that WRI is associated not only with lower affective well-being but also with subsequent energy depletion and cognitive overload (Sonnentag & Zijlstra, 2006; Perko et al., 2017; Jia et al., 2024). In post-holiday contexts, WRI emerges as a key emotional marker of how employees react to abrupt changes in work intensity and insufficient resource restoration.

The direct consequence of such irritation is an elevated need for recovery (NR). NR represents the psychological and physiological urge to recuperate from work-induced effort and strain (Van Veldhoven & Broersen, 2003). It captures an individual’s subjective experience of fatigue, reduced concentration, and unwillingness to exert further effort until adequate rest is achieved (Demerouti et al., 2009). Following extended breaks, employees may find that renewed work demands arrive too quickly or intensely, limiting the restorative benefits of the holiday period. As a result, the return to work is accompanied by heightened NR, which in turn undermines the individual’s capacity for sustained energy and engagement (André & Baumeister, 2023). In line with this, we propose the following hypothesis:

**H1.**  *Work-related irritation is associated with increased need for recovery.*

The final link in this post-holiday adjustment chain concerns workplace vigor (WV). WV is a core dimension of work engagement, defined as high levels of energy, mental resilience, and persistence in the face of challenges (Schaufeli et al., 2002; Shirom, 2003a). Vigor reflects an employee’s readiness to invest effort and enthusiasm into work activities (Shirom, 2007, 2011). However, high levels of NR have been repeatedly shown to inhibit WV by sapping internal resources necessary for cognitive activation and motivational intensity (Balk et al., 2021; Zijlstra et al., 2014). Employees who resume work while still experiencing elevated recovery needs, possibly as a result of lingering fatigue or insufficient resource replenishment, are less able to sustain vigor, which may result in lower engagement and impaired performance. Therefore, we propose the following hypothesis:

**H2.**  *Need for recovery is expected to negatively affect workplace vigor.*

### 2.2. The Mediating Role of Need for Recovery

Returning to work after an extended holiday presents employees with a distinct psychological challenge that may temporarily disrupt their emotional and cognitive readiness for work. While the previous section outlined how accumulated strain can trigger irritability and energy loss, the current section further explores the underlying psychological mechanism that transmits these emotional disturbances into reduced performance, namely, the employee’s need for recovery. Drawing on COR theory (Hobfoll, 1989), recovery is understood as the process through which individuals restore valued personal resources such as energy, attention, and emotional stability that have been depleted by environmental demands (Trougakos & Hideg, 2009). When resource replenishment is insufficient, especially after a period of rest such as a holiday, employees may experience persistent fatigue, reduced concentration, and diminished motivation, making it difficult to fully invest in their tasks.

Empirical studies have consistently demonstrated that emotional strain at work, especially after time away from job demands, leads to a heightened sense of recovery need (Sonnentag et al., 2010; Simbula et al., 2019). When individuals return from holiday without fully replenishing their personal resources, they may find the work environment cognitively overwhelming and emotionally taxing. This imbalance often triggers a strong psychological impulse to withdraw, mentally disengage, or slow down, reflecting an internal effort to preserve remaining resources. Research shows that recovery need tends to increase when external demands rise rapidly and the available resources are insufficient to meet those demands (Zijlstra et al., 2014; Sonnentag & Fritz, 2015). In this context, recovery needs serve as a motivational signal, prompting individuals to reduce workload intensity, postpone full engagement, or seek supportive mechanisms that aid the restoration process.

The consequences of unmet recovery needs extend beyond temporary discomfort. Prior studies have found that employees with elevated recovery demands tend to display significantly lower levels of vigor (Sonnentag & Niessen, 2008; Sonnentag et al., 2012; Bennett et al., 2018), a key component of work engagement defined by energy, mental resilience, and persistence (Shirom, 2003a). From this perspective, recovery need becomes not merely an outcome of emotional disruption but a mediator that links emotional strain to behavioral and attitudinal disengagement at work. The mediational mechanism can be interpreted through resource-based theories: When employees experience psychological fatigue but cannot afford or are not permitted recovery time, their work-related energy and enthusiasm gradually erode. This leads to a downward spiral where emotional strain, recovery demand, and performance impairments reinforce each other. Building on these theoretical and empirical foundations, we formulate the following hypothesis:

**H3.**  *Work-related irritation is expected to negatively affect workplace vigor (H3a), and this relationship is expected to be negatively mediated by need for recovery (H3b).*

### 2.3. The Moderating Role of Boundary Strength at Home and Perceived Organizational Support

#### 2.3.1. Boundary Strength at Home as a Moderator

According to COR theory, the ability to protect and restore personal resources is influenced by the environmental structures that enable or constrain access to those resources (Hobfoll, 1989, 2001). In the context of post-holiday adjustment, boundary strength at home (BSH) plays a pivotal role in shaping how well employees recover during the holiday period and how they respond to work demands upon return. BSH refers to the extent to which individuals are able to maintain clear and impermeable distinctions between their home and work roles while outside of work (Hecht & Allen, 2009). Employees with stronger home boundaries are more likely to protect their leisure time from work-related intrusions and achieve deeper psychological detachment during time off (Mellner, 2016; Sonnentag, 2012).

Research suggests that psychological detachment during nonwork time is essential for recovery, especially after periods of stress or sustained work effort (Sonnentag & Fritz, 2015; Demerouti et al., 2009). When boundaries are strong, employees are better able to engage in recovery experiences such as relaxation, low-effort activities, and emotional regulation (Kinnunen et al., 2016; Bennett et al., 2016). Consequently, these individuals enter the post-holiday transition period with greater emotional readiness and restored personal resources, making them less susceptible to the negative effects of work-related irritation. In contrast, individuals with weak boundaries may continue to think about work during holidays or even engage in job-related activities, thereby undermining the restorative function of the break (Sonnentag & Braun, 2013).

Empirical findings further support that boundary management practices significantly influence recovery quality. For instance, research has shown that strong segmentation preferences (i.e., the desire and ability to keep work and home domains distinct) are positively related to well-being and negatively associated with strain and burnout (Althammer et al., 2021; Haun et al., 2022; Jensen, 2023). These findings highlight the buffering effect of firm boundaries in protecting employees against emotional exhaustion and impaired functioning. Based on this reasoning, we propose the following hypothesis:

**H4.**  *Boundary strength at home moderates the positive relationship between work-related irritation (WRI) and need for recovery (NR), such that employees with higher boundary strength at home will experience a weaker positive relationship between WRI and NR.*

#### 2.3.2. Perceived Organizational Support as a Moderator

While boundary strength at home serves to enhance recovery during nonwork periods, perceived organizational support (POS) becomes particularly salient when employees return to work and face renewed demands. POS refers to employees’ general beliefs that their organization values their contributions and cares about their well-being (Eisenberger et al., 1986). A growing body of literature has shown that POS plays a critical buffering role in helping employees manage stress, reduce emotional exhaustion, and remain engaged even under challenging work conditions (Rhoades & Eisenberger, 2002; Caesens et al., 2017).

From a resource conservation perspective, returning to work after a holiday is often accompanied by weakened psychological readiness, increased recovery need, and heightened sensitivity to environmental cues. In such contexts, organizational support can serve as a compensatory resource that facilitates smoother re-engagement. According to COR theory, POS represents a key socio-emotional resource provided by the organization, which can prevent further loss cycles and reinforce employees’ psychological resilience. Specifically, high POS provides employees with a sense of psychological safety and reassurance, which can reduce anxiety and mitigate the energy-draining effects of unresolved recovery needs (Serhan et al., 2025). Employees who perceive strong organizational support may be more motivated to activate personal resources, reinterpret fatigue as manageable, and maintain effort despite incomplete recovery.

Empirical studies further support the buffering role of POS. For example, research has shown that POS moderates the relationship between stressors and burnout and enhances the effects of job resources on engagement and well-being (Kurtessis et al., 2017). When employees perceive their organization as supportive, they are more likely to experience less depletion in motivation and vitality, even when their recovery needs are high (Schaufeli & Bakker, 2004; Kurtessis et al., 2017; Caesens & Stinglhamber, 2014). This suggests that POS may attenuate the negative impact of recovery-related strain on energy-intensive work behaviors, such as vigor. Based on these arguments, we propose the following hypothesis:

**H5.**  *Perceived organizational support moderates the negative relationship between need for recovery (NR) and workplace vigor (WV), such that employees with greater perceived organizational support will experience a weaker negative relationship between NR and WV.*

### 2.4. Moderated Mediation Effect of BSH and POS

The process of returning to work after a holiday involves complex emotional, cognitive, and behavioral adjustments. As outlined earlier, employees often experience elevated recovery needs in response to post-holiday irritation, which in turn undermines their ability to maintain workplace vigor. However, this indirect pathway is unlikely to be uniform across individuals. According to COR theory, the extent to which emotional strain and energy depletion occur depends not only on situational demands but also on the availability and effectiveness of personal and organizational resources to manage those demands (Hobfoll, 1989; Hobfoll et al., 2018).

This study posits that the indirect relationship between post-holiday emotional disturbance and workplace vigor is jointly moderated by BSH and POS. BSH operates primarily during the holiday period, enabling employees to engage in deep recovery by minimizing cognitive and behavioral intrusion from work (Haun et al., 2022). In contrast, POS functions during the return-to-work phase by providing reassurance, validation, and psychological safety (Appelbaum et al., 2022). These two moderators operate at different stages but together form a dual-buffering system that shapes the extent to which post-holiday irritation leads to recovery demand and reduced vigor.

When both BSH and POS are high, employees are likely to return to work more refreshed and psychologically prepared. As a result, any residual recovery needs are less likely to disrupt their work engagement. In contrast, employees with low BSH and low POS may struggle at both stages of the adjustment process. They may fail to achieve meaningful recovery during the holiday and also receive limited support upon returning to work. In such cases, the indirect effect of emotional strain on workplace vigor is expected to be most pronounced. This logic is supported by studies showing that the combined effect of recovery-facilitating conditions and organizational resources significantly predicts well-being and performance outcomes (ten Brummelhuis & Bakker, 2012). Therefore, we propose the following hypothesis:

**H6.**  *Boundary strength at home (BSH) and perceived organizational support (POS) jointly moderate the indirect effect of work-related irritation on workplace vigor via need for recovery, such that the indirect effect is weakest when both BSH and POS are high.*

**Figure 1:**
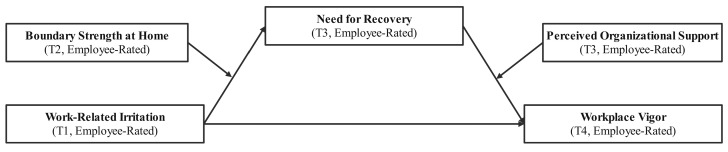
Conceptual model.

Accordingly, the conceptual framework of this study is illustrated in Figure 1.

## 3. Materials and Methods

### 3.1. Design and Sample

The study employs a four-wave time-lagged design to examine the short-term impact of holiday blues syndrome on workplace vigor. Data were collected via online surveys from employees across diverse industries and job roles in multiple provinces of China. The research spanned approximately one month, beginning on 27 January 2025, with each measurement wave conducted at weekly intervals. At Time 1 (27 January 2025; the last day before the Chinese Lunar New Year holiday), participants completed demographic information and reported their levels of work-related irritation (WRI). At Time 2 (4 February 2025; the last day of the holiday), participants assessed the strength of their home boundaries (BSH). At Time 3 (11 February 2025; the final working day of the first post-holiday week), participants reported their need for recovery (NR) and perceived organizational support (POS). At Time 4 (18 February 2025; the midpoint of the second post-holiday workweek), participants evaluated their workplace vigor (WV).

### 3.2. Questionnaire Design and Survey Measures

To align with the time-lagged design of the study, we developed four independent questionnaires, each administered exclusively during its respective wave of data collection. The first questionnaire included a brief introduction explaining the study’s objectives and time-lagged design, informing participants that they would be invited to complete follow-up surveys over the next month. To maintain anonymity and ensure accurate data matching across all four waves, participants generated a unique response ID by combining their initials with the last four digits of their phone number. The study utilized a five-point Likert scale, with the sources of the measurement scales and example items provided below.

#### 3.2.1. Work-Related Irritation

We measured work-related irritation using the 8-item scale developed by Mohr et al. (2006), which assesses psychological strain in work contexts (Cronbach’s α = 0.91). Example items include “Before the holiday, I found it difficult to relax and kept thinking about work” and “During the week before the holiday, I became easily irritated by interruptions from colleagues or family members”.

#### 3.2.2. Boundary Strength at Home

We assessed boundary strength at home using the 8-item subscale of the work–nonwork boundary strength scale developed by Hecht and Allen (2009), which evaluates the strength of work–nonwork boundaries during the holiday (Cronbach’s α = 0.94). Example items include “I maintain clear boundaries between my work and personal life during the holiday” and “My personal time is fully dedicated to non-work activities throughout the holiday”.

#### 3.2.3. Need for Recovery

We assessed the need for recovery using the 11-item scale developed by Van Veldhoven and Broersen (2003), which measures employees’ physical and mental recovery needs during the transition back to work following holidays (Cronbach’s α = 0.95). Example items include “After a workday, I find it difficult to fully relax” and “I often feel extremely exhausted at the end of the workday, preventing me from participating in other activities”.

#### 3.2.4. Perceived Organizational Support

We evaluated perceived organizational support using the 10-item scale developed by Eisenberger et al. (1986), which measures employees’ perceptions of organizational care and support during the transition back to work after holidays (Cronbach’s α = 0.93). Example items include “My organization provides appropriate help and resources when I encounter difficulties adapting to work after the holiday” and “My organization genuinely cares about my well-being and takes measures to support my recovery and adjustment”.

#### 3.2.5. Workplace Vigor

We assessed workplace vigor using the 14-item scale developed by Shirom (2003a), which includes three subdimensions: physical strength, cognitive liveliness, and emotional energy (Cronbach’s α = 0.94). Example items include “I feel full of energy when returning to work after the holiday” (physical strength), “I can quickly regain a clear state of mind after the holiday” (cognitive liveliness), and “I continue to show empathy and teamwork even immediately after returning to work” (emotional energy).

#### 3.2.6. Control Variables

Existing literature suggests that employees’ recovery needs and workplace vigor can be influenced by several individual and work-related characteristics, such as age, job role, overtime work, exercise frequency, daily sleep duration, and industry type (e.g., Sonnentag & Fritz, 2007). These variables were included as controls because they may confound the observed relationships among the focal constructs by influencing baseline energy levels, strain susceptibility, or recovery capacity. For example, older employees may show better resilience to job stress but tend to have lower work vigor (Mauno et al., 2013). Employees in managerial roles may experience higher work-related irritation and recovery needs due to increased responsibilities (Burke et al., 2009; Burke & El-Kot, 2009). Long working hours and overtime are associated with higher stress and fatigue, leading to increased recovery needs (Folkard & Tucker, 2003). Additionally, employees with regular exercise habits and sufficient sleep duration often demonstrate stronger recovery capacity and workplace vigor (Sonnentag et al., 2017; Wiegelmann et al., 2023). Industry type may also play a significant role, as different industries have varying job demands and work environments, which could impact employees’ recovery processes and workplace vigor (Zhang et al., 2024; Selander et al., 2025).

### 3.3. Data Collection and Screening

We adopted a network sampling approach by leveraging one author’s professional contacts to recruit participants. Invitations were sent to approximately 500 employees from a wide range of industries via WeChat, with 465 consenting to participate. Responses were submitted using the participant-generated IDs described in the initial questionnaire to ensure consistency across waves.

Each wave of data collection was launched precisely on the designated measurement date. The first survey was distributed on 27 January 2025, followed by subsequent waves on 4 February, 11 February, and 18 February. Response rates remained consistently high across waves, yielding 403, 390, 381, and 372 valid responses, respectively. To maintain participant engagement throughout the month-long process, brief reminders were sent before each wave, and the survey links were kept active for 48 h.

Before proceeding with statistical analyses, the data underwent cleaning and screening. First, we matched responses from all four waves using the unique response IDs generated by the respondents; this yielded an initial pool of 372 complete responses. The dataset was then checked for missing values and careless responses. Sixteen cases with missing data and improper responses were identified and removed. Subsequently, seven cases were excluded as statistical outliers based on Cook’s distance values exceeding the threshold of 1 (Stevens, 2002). These outliers showed extreme response patterns and could have distorted regression estimates if retained. This process resulted in a final sample of 349 valid responses. The exclusion of these cases did not materially affect the direction or significance of the results, suggesting minimal risk of bias. The demographic profile of these respondents is presented in Table 1.

## 4. Results

We conducted statistical analyses with SPSS 26.0 and Mplus 8.3. Descriptive statistics, correlation analysis, reliability, and validity assessments were performed with SPSS 26.0, whereas confirmatory factor analysis and hypothesis testing were carried out using Mplus 8.3.

### 4.1. Descriptive Statistics and Correlations

Table 2 presents the means, standard deviations, and intercorrelations among all study variables. As shown in the table, work-related irritation is positively correlated with need for recovery (r = 0.476, *p* < 0.001), and both are negatively correlated with workplace vigor (r = −0.311 and −0.409, respectively, both *p* < 0.001), providing preliminary support for both the hypothesized direct effect and the mediating relationship.

The table also reports the composite reliability (CR), average variance extracted (AVE), and the square root of AVE for each latent construct. The CR values of all constructs exceed the recommended threshold of 0.80 (Fornell & Larcker, 1981), indicating strong internal consistency reliability. Moreover, the square root of AVE for each variable is greater than its correlations with other constructs, demonstrating adequate discriminant validity according to Fornell and Larcker’s criterion.

In addition, descriptive analysis showed that the average age of participants was approximately 37 years, with most respondents falling in the 26–45 age range. On average, participants reported working overtime about 15 h per week, exercising 2–3 times per week, and sleeping between 6 and 7 h per day. These variables were included as statistical controls in subsequent analyses to account for individual differences in demographic and lifestyle factors.

### 4.2. Measurement Model

We conducted confirmatory factor analysis (CFA) to evaluate the construct validity of the study variables, including work-related irritation, need for recovery, workplace vigor, boundary strength at home, and perceived organizational support. As shown in Table 3, the hypothesized five-factor model demonstrated the best fit to the data (χ^2^ = 1887.137, df = 1214, χ^2^/df = 1.554, RMSEA = 0.040, CFI = 0.945, TLI = 0.942, SRMR = 0.040), indicating adequate discriminant validity among the focal constructs. To further test for potential common method bias, we specified a six-factor model including an unmeasured latent method factor (Model 6). The fit indices for this model (χ^2^ = 1882.530, χ^2^/df = 1.557, RMSEA = 0.040, CFI = 0.944, TLI = 0.941, SRMR = 0.040) were comparable to those of the original five-factor model and did not show significant improvement. These results suggest that common method bias is unlikely to be a major concern in this study. In addition, to strengthen evidence for construct validity, we provide standardized factor loadings in Table A1, with most items exceeding the 0.70 threshold. Table A2 presents cross-loadings, confirming that each item loads more strongly on its designated construct than on others, thus supporting discriminant validity. Table A1 and Table A2 are presented in Appendix A.

### 4.3. Hypothesis Testing

This study tested the proposed hypotheses while accounting for all relevant control variables (see Section 3.2.6). Table 4 presents the standardized path coefficients for the main study variables derived from the structural equation model. Among the control variables, only exercise frequency was significantly associated with workplace vigor (β = −0.095, *p* < 0.05), suggesting that lower levels of physical activity during the holiday were linked to reduced post-holiday energy. The other control variables did not show significant associations with either need for recovery or workplace vigor.

Hypothesis 1 proposes that work-related irritation (WRI) is positively associated with increased need for recovery (NR). As shown in Table 4, WRI is significantly positively associated with NR (β = 0.400, SE = 0.060, *p* < 0.001), supporting Hypothesis 1. The positive coefficient indicates that employees who experience greater emotional strain prior to the holiday tend to report stronger recovery needs during the post-holiday adjustment period.

Hypothesis 2 proposes that the need for recovery (NR) negatively affects workplace vigor (WV). As shown in Table 4, NR is significantly negatively associated with WV (β = −0.287, SE = 0.065, *p* < 0.001), supporting Hypothesis 2. This result suggests that employees who report higher recovery needs following a holiday tend to experience lower levels of energy and enthusiasm at work.

Hypothesis 3 proposed that (a) work-related irritation (WRI) would negatively affect workplace vigor (WV), and (b) this relationship would be mediated by need for recovery (NR). As shown in Table 4, the direct path from WRI to WV was not significant (β = −0.066, SE = 0.075, *p* > 0.05), providing no support for Hypothesis 3a. However, WRI was significantly positively associated with NR (β = 0.400, SE = 0.060, *p* < 0.001), and NR was significantly negatively associated with WV (β = −0.287, SE = 0.065, *p* < 0.001; see Table 4), suggesting a possible mediating relationship.

To further test the mediation effect, we conducted a bias-corrected bootstrap analysis with 5000 resamples. As reported in Table 5, the indirect effect of WRI on WV via NR was significant (β = −0.115, SE = 0.030, 95% CI [−0.187, −0.067]), as the confidence interval did not include zero. Therefore, Hypothesis 3b is supported, while Hypothesis 3a is not. This pattern indicates a full mediation effect, in which work-related irritation influences workplace vigor only indirectly through recovery need. A possible explanation is that employees’ emotional strain before the holiday may dissipate during time off and only affect post-holiday vigor through accumulated recovery demands.

**Figure 2:**
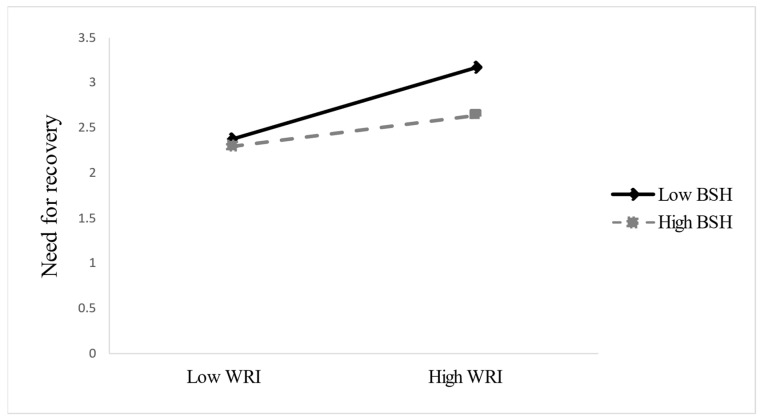
BSH moderates the relationship between WRI and NR. BSH = boundary strength at home; WRI = work-related irritation; NR = need for recovery.

Hypothesis 4 proposes that boundary strength at home (BSH) moderates the positive relationship between work-related irritation (WRI) and need for recovery (NR), such that this relationship is weaker when BSH is high. As shown in Table 4, the interaction between WRI and BSH is significant (β = −0.231, SE = 0.045, *p* < 0.001), supporting Hypothesis 4. Figure 2 illustrates that WRI is more strongly associated with NR when BSH is low (β = 0.400), whereas the association is weaker when BSH is high (β = 0.169). This pattern suggests that strong home boundaries help buffer the emotional spillover from work irritation, thereby reducing recovery demands after the holiday. The difference in slopes (Δβ = 0.231) reflects a meaningful moderation effect.

**Figure 3:**
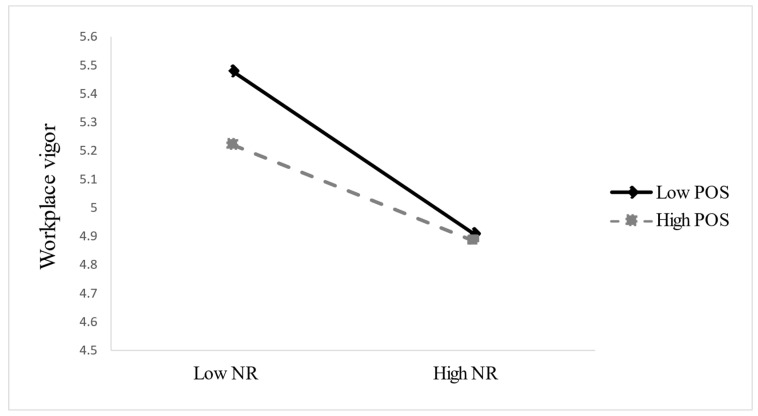
POS moderates the relationship between NR and WV. POS = perceived organizational support; NR = need for recovery; WV = workplace vigor.

Hypothesis 5 posits that perceived organizational support (POS) moderates the negative relationship between need for recovery (NR) and workplace vigor (WV), such that this relationship is weaker when POS is high. As shown in Table 4, the interaction between NR and POS is significant (β = 0.118, SE = 0.052, *p* < 0.05), supporting the hypothesized buffering effect. Figure 3 illustrates this pattern: When POS is low, NR is strongly negatively associated with WV (β = −0.287), whereas the association is weaker under high POS (β = −0.169), yielding a slope difference of Δβ = 0.118. This suggests that employees who perceive higher organizational support are less vulnerable to recovery-related declines in vigor. While the visual contrast appears modest under high NR conditions, this may reflect compensatory effort among high-POS employees or sample distribution effects. We further discuss this phenomenon in the following section.

Hypothesis 6 proposes that boundary strength at home (BSH) and perceived organizational support (POS) jointly moderate the indirect effect of work-related irritation (WRI) on workplace vigor (WV) through need for recovery (NR), such that the indirect effect is weakest when both BSH and POS are high. As shown in Table 6, the indirect effect of WRI on WV via NR is strongest when both BSH and POS are low (estimate = −0.256, SE = 0.062, 95% CI [−0.387, −0.145]) and gradually weakens as either BSH or POS increases. Specifically, the conditional indirect effects are significant but attenuated under conditions of either high BSH or high POS (estimate = −0.106 and −0.069, respectively) and become weakest when both moderators are high (estimate = −0.029, SE = 0.019, 95% CI [−0.081, −0.001]). These results indicate that strong boundary strength at home and high POS jointly buffer the indirect path from WRI to WV via NR, thereby supporting Hypothesis 6.

## 5. Discussion

This study employed a four-wave time-lagged design to examine how employees’ emotional responses and recovery needs influence their work vitality after returning from holidays. Based on COR theory, the findings support the proposed moderated mediation model, confirming the indirect effect of pre-holiday emotional strain on post-holiday workplace vigor through recovery need. The results also highlight the significance of both personal boundary management and organizational support in helping preserve and restore psychological resources and sustaining employee energy during the post-holiday adjustment period.

### 5.1. Theoretical Implications

Our study contributes to the academic literature in four ways. First, this study extends COR theory (Hobfoll, 1989; Hobfoll et al., 2018) by applying it to the context of post-holiday adjustment. COR theory posits that individuals are motivated to acquire, retain, and protect valued resources, and stress arises when these resources are lost or insufficiently restored (Hobfoll, 1989; Halbesleben et al., 2014). In the case of extended breaks, such as public holidays, employees are expected to recover depleted psychological resources. However, our findings suggest that when pre-holiday emotional strain is high and recovery is incomplete, resource replenishment is impaired, leading to diminished workplace vigor. This application broadens the utility of COR theory by illustrating how recovery failure during scheduled nonwork periods can threaten resource balance and hinder short-term work reengagement.

Second, we contribute to the recovery literature by highlighting the dynamic nature of post-holiday adjustment and by positioning the need for recovery (NR) as a novel mediator. While prior studies have mainly focused on daily stressors and short-term recovery (Sonnentag & Geurts, 2009; Sonnentag et al., 2017), less is known about recovery after extended breaks. Our findings suggest that pre-holiday emotional strain influences employees’ recovery needs, which in turn affect their ability to reengage with work. Although Hypothesis 3a was not supported, this may reflect a time lag between irritation and vigor measurements, implying that the effect is indirect and short-lived—consistent with prior evidence on anticipatory stress dissipation (Sonnentag & Fritz, 2015; Bolger & Zuckerman, 1995). Furthermore, while NR is typically treated as an outcome in occupational health research, recent studies (e.g., Rus et al., 2022; Jin et al., 2023) have begun to view it as a dynamic mediator linking stressors to motivational outcomes. Our model extends this perspective and responds to calls for integrating recovery processes into models of work engagement (Sonnentag & Niessen, 2008; Kinnunen et al., 2011), offering a more nuanced understanding of how short-term strain shapes post-holiday functioning.

Third, we contribute to the literature by introducing a moderation framework that integrates both personal and organizational resources. While previous studies have examined the buffering effect of perceived organizational support (Eisenberger et al., 1986; Rhoades & Eisenberger, 2002) or boundary management strategies (Kreiner et al., 2009) separately, few have explored their interactive effects in a post-holiday context. By showing that the combination of strong home boundaries and high organizational support weakens the indirect pathway from emotional strain to diminished vigor, our study provides a more integrated view of how employees conserve and mobilize resources across multiple domains to sustain energy following periods of strain (ten Brummelhuis & Bakker, 2012; Volman et al., 2013).

Finally, our study contributes to the growing body of research that shifts focus from burnout to positive psychological states such as vigor. While burnout and emotional exhaustion have been dominant in stress and recovery research (Maslach et al., 2001; Shirom, 2003b), recent scholarship emphasizes the need to understand how energy, motivation, and resilience are preserved or enhanced (Liu & Boyatzis, 2021; Chan et al., 2022; Kuntz, 2024). By placing workplace vigor as the outcome, we enrich the theoretical conversation on how employees sustain energy through effective resource management, particularly in the face of renewed demands after periods of rest and detachment.

In addition to these contributions, we also note a visually unexpected pattern in Figure 3, where the difference in workplace vigor between high- and low-POS groups appears relatively small under high need for recovery. Although the overall interaction effect supports Hypothesis 5, this result invites further theoretical reflection. One possible explanation is that employees with high perceived organizational support may exert compensatory effort when experiencing resource depletion, pushing themselves to maintain performance despite fatigue, which may paradoxically suppress their perceived vigor (Hobfoll, 1989; Halbesleben & Bowler, 2007). In contrast, low-POS employees may adopt resource-conserving strategies or psychologically disengage, thus reporting slightly higher levels of residual energy. Another possibility is that employees in low-POS environments may have developed stronger self-regulatory mechanisms to cope with inadequate support, such as goal pacing or emotional detachment (Hobfoll, 1989; De Cuyper et al., 2011). These patterns suggest that the relationship between organizational support and vigor may be more complex under high-stress conditions and deserves closer examination in future research. Moreover, such compensatory or adaptive mechanisms may differ across employee groups (e.g., by tenure, job role, or self-regulation capacity), warranting future research into segment-specific variations in how BSH and POS jointly shape recovery outcomes.

### 5.2. Practical Implications

This study provides several practical implications for organizations aiming to improve employee adjustment and performance in the post-holiday period. As the findings demonstrate, the post-holiday transition is not only a logistical shift but also a psychologically taxing process for many employees. Therefore, organizations must take a more proactive and recovery-informed approach to human resource management during this sensitive period.

First, organizations should consider adjusting post-holiday task structures and workflows to accommodate employees’ recovery needs. As shown in our results, employees may experience elevated emotional strain and recovery demands after extended holidays, which can hinder their energy and engagement. Managers are encouraged to implement gradual workload ramp-up strategies, such as postponing high-pressure assignments, prioritizing low-complexity tasks, and limiting intensive meetings during the first week back to work (Sonnentag & Fritz, 2015; Binnewies et al., 2009). Furthermore, flexible scheduling options (e.g., staggered deadlines, hybrid work arrangements, or lighter project timelines) can support a smoother return to work and reduce adaptation costs (Allen et al., 2013; Sardeshmukh et al., 2012). These strategies not only benefit employee well-being but may also improve long-term performance quality.

Second, companies should promote clear and flexible boundary management practices, especially before and after holiday periods. Since boundary strength at home plays a significant role in facilitating psychological detachment and post-holiday recovery, organizations can offer training, coaching, or digital tools to help employees manage work–nonwork boundaries more effectively (Kreiner et al., 2009). For example, managers can communicate clear expectations around response time, discourage unnecessary work communications during holidays, and create shared norms that respect personal time. A boundary-supportive work climate not only protects recovery experiences but also enhances perceived autonomy and promotes sustainable engagement (Derks et al., 2014).

Third, our findings highlight the importance of perceived organizational support as a protective factor during the post-holiday adjustment period. Organizations should actively signal care and appreciation for employees during this phase through targeted resources such as mental health programs, team debriefing activities, return-to-work onboarding sessions, or short-term incentives (Eisenberger & Stinglhamber, 2011; Rhoades & Eisenberger, 2002). Proactive communication, supportive supervision, and structured peer mentoring can foster psychological safety and mitigate the motivational consequences of elevated recovery needs. Importantly, this study suggests that perceived organizational support is most effective when combined with strong individual boundary management, pointing to the need for integrated support systems that align personal strategies with organizational practices. Moreover, organizations should be aware that perceived support, while generally beneficial, may sometimes lead employees to engage in compensatory efforts that unintentionally deplete their energy. This suggests that support initiatives should be coupled with explicit recovery allowances and realistic performance expectations to avoid unintended strain, especially among highly committed employees (Sonnentag & Fritz, 2015; ten Brummelhuis & Bakker, 2012).

Finally, by focusing on workplace vigor rather than just strain or burnout, our study encourages organizations to prioritize positive energy management as part of their post-holiday reintegration efforts. Instead of merely aiming to reduce fatigue or prevent burnout, managers should design interventions that stimulate intrinsic motivation and positive affect, such as team goal-setting workshops, energizing check-ins, or public recognition of effort and progress (Bakker & Demerouti, 2008; Steegh et al., 2025; Delavallade, 2021). These proactive strategies can help employees re-engage more quickly and maintain vitality over time, ultimately contributing to a healthier, more resilient, and more productive workforce.

### 5.3. Limitations and Future Research

Despite its contributions, this study has several limitations that suggest avenues for future research. First, this study employed a four-wave time-lagged design to reduce common method bias and establish temporal precedence among variables. While this approach enhances causal inference to some extent, it does not constitute a full longitudinal or multi-level design. Future research could consider adopting repeated measurements of key variables (e.g., recovery need and workplace vigor) over multiple time points or implementing multi-level designs that account for nested structures such as teams or work units.

Second, the study was conducted in the context of the Chinese Lunar New Year, which is not only the longest public holiday in China but also carries strong cultural and social significance. While this setting provided a unique opportunity to observe pronounced shifts in employees’ resource states and recovery dynamics, the generalizability of the findings may be limited to similar cultural contexts. Future studies could replicate the model in different countries, organizational settings, or holiday types to explore cultural variations in post-holiday adjustment mechanisms.

Third, although the study considered both personal factors and organizational conditions as moderators, it did not explicitly measure cross-level interactions or team-level influences. Social norms, leadership style, or team climate may play an important role in shaping how individuals recover and reengage after holidays. Future research could employ multilevel modeling to examine how shared team practices and organizational policies interact with personal factors to influence recovery dynamics.

Finally, this study focused solely on workplace vigor as the primary positive outcome. While vigor is a key dimension of employee well-being, future studies could extend the model to include additional outcomes such as creativity, voice behavior, presenteeism, or long-term job performance. Examining a broader set of behavioral and attitudinal outcomes would provide a more comprehensive understanding of how post-holiday transitions influence employees and organizations.

## 6. Conclusions

This study explored how employees psychologically adjust after holidays and how emotional strain, recovery needs, and contextual resources jointly influence workplace vigor. Drawing on COR theory, we proposed and tested a moderated mediation model using a four-wave time-lagged design around the Chinese Spring Festival. The findings confirmed that work-related irritation experienced before holidays is significantly linked to higher recovery needs, which are in turn related to lower levels of post-holiday vigor. Moreover, boundary strength at home and perceived organizational support served as effective buffers that helped mitigate this negative pathway. This study contributes to a deeper understanding of how resource loss and replenishment processes shape employee functioning after extended breaks and provides actionable insights for supporting employees’ energy and performance in the aftermath of recovery periods.

## Figures and Tables

**Table 1:** Sociodemographic characteristics of the sample.

Characteristic	Category	Frequency (n)	Percentage (%)
Age	25 years and below	93	26.6
	26–35 years	128	36.7
	36–45 years	37	10.6
	46–55 years	53	15.2
	56 years and above	38	10.9
Industry type	High-intensity and shift-based industries (e.g., transport, healthcare, manufacturing, and utilities)	5	1.4
	Knowledge-intensive and high-responsibility industries (e.g., IT, finance, education, and legal)	213	61
	Service-oriented and customer-facing industries (e.g., retail, hospitality, and media)	122	35
	Government and public administration (e.g., public sector and non-profits)	6	1.7
	Other	3	0.9
Job role	Service position	39	11.2
	Managerial position	156	44.7
	Technical position	95	27.2
	Sales position	37	10.6
	Frontline operations position	16	4.6
	Other	6	1.7
Overtime work	Rarely	25	7.2
	Occasionally	104	29.8
	Frequently	128	36.7
	Almost every day	92	26.4
Exercise frequency	Never	21	6
	1–2 times per week	110	31.5
	3–5 times per week	112	32.1
	Almost every day	106	30.4
Daily sleep duration	Less than 5 h	12	3.4
	5–6 h	127	36.4
	7–8 h	128	36.7
	9 h or more	82	23.5

**Table 2:** Means, standard deviations, and correlations among all variables.

Variable	Mean	SD	1	2	3	4	5
1. Work-related irritation	3.491	0.806	1				
2. Need for recovery	3.191	0.918	0.476 ***	1			
3. Workplace vigor	3.464	0.861	−0.311 ***	−0.409 ***	1		
4. Boundary strength at home	3.479	1.011	−0.440 ***	−0.492 ***	0.388 ***	1	
5. Perceived organizational support	3.448	0.934	0.426 ***	0.411 ***	−0.326 ***	−0.307 ***	1
CR	-	-	0.913	0.945	0.938	0.944	0.926
AVE	-	-	0.570	0.612	0.519	0.680	0.556
Square root of AVE	-	-	0.755	0.782	0.720	0.825	0.746

Notes: CR = composite reliability; AVE = average variance extracted. *** *p* < 0.001. Sample size: N = 349.

**Table 3:** Results of confirmatory factor analysis (CFA).

Models	χ^2^	df	χ^2^/df	RMSEA	CFI	TLI	SRMR
Model 1: Five-factor model (including 1, 2, 3, 4, 5)	1887.137	1214	1.554	0.040	0.945	0.942	0.040
Model 2: Four-factor model (including 1 + 2, 3, 4, 5)	2982.454	1218	2.449	0.064	0.855	0.848	0.070
Model 3: Three-factor model (including 1 + 2 + 3, 4, 5)	4909.621	1221	4.021	0.093	0.696	0.683	0.107
Model 4: Two-factor model (including 1 + 2 + 3 + 4, 5)	6342.963	1223	5.186	0.110	0.578	0.560	0.117
Model 5: One-factor model (including 1 + 2 + 3 + 4 + 5)	7714.924	1224	6.303	0.123	0.465	0.443	0.133
Model 6: Unmeasured latent method factor model	1882.530	1209	1.557	0.040	0.944	0.941	0.040

Notes: 1 = work-related irritation; 2 = need for recovery; 3 = workplace vigor; 4 = boundary strength at home; 5 = perceived organizational support. Model 6 = Model 1 + an unmeasured latent method factor. All items were merged into one method factor.

**Table 4:** Standardized path coefficients from the structural equation model.

Main Variables	Need for Recovery		Workplace Vigor	
	Estimate	SE	Estimate	SE
Work-related irritation (WRI)	0.400 ***	0.060	−0.066	0.075
Boundary strength at home (BSH)	−0.309 ***	0.043		
WRI × BSH	−0.231 ***	0.045		
Need for recovery (NR)			−0.287 ***	0.065
Perceived organizational support (POS)			−0.142 *	0.066
NR × POS			0.118 *	0.052
R^2^	0.373		0.221	

Notes: * *p* < 0.05, *** *p* < 0.001. Sample size: N = 349.

**Table 5:** Mediation effects of NR on the relationship between WRI and WV.

Type of Effect	Path	Estimate	SE	Bootstrap 95% CI	
				LLCI	ULCI
Direct effect	WRI→WV	−0.066	0.075	−0.222	0.078
Indirect effect	WRI→NR→WV	−0.115	0.030	−0.187	−0.067

Notes: WRI = work-related irritation; NR = need for recovery; WV = workplace vigor.

**Table 6:** Conditional indirect effects of WRI on WV via NR at different levels of BSH and POS.

BSH × POS Level	Path	Estimate	SE	Bootstrap 95% CI	
				LLCI	ULCI
Low BSH (−1 SD), Low POS (−1 SD)	WRI→NR→WV	−0.256	0.062	−0.387	−0.145
Low BSH (−1 SD), High POS (+1 SD)	WRI→NR→WV	−0.106	0.045	−0.196	−0.023
High BSH (+1 SD), Low POS (−1 SD)	WRI→NR→WV	−0.069	0.037	−0.153	−0.002
High BSH (+1 SD), High POS (+1 SD)	WRI→NR→WV	−0.029	0.019	−0.081	−0.001

Notes: WRI = work-related irritation; NR = need for recovery; WV = workplace vigor; BSH = boundary strength at home; POS = perceived organizational support.

## Data Availability

The original contributions presented in this study are included in the article. Anonymized data may be made available from the corresponding author upon reasonable request and with approval from the institutional ethics committee. Further inquiries can be directed to the corresponding author.

## Appendix A

**Table A1:** Factor loadings of measurement items.

Variables	Items	Factor Loadings
Work-related irritation	WRI1	0.807
	WRI2	0.780
	WRI3	0.839
	WRI4	0.728
	WRI5	0.699
	WRI6	0.820
	WRI7	0.817
	WRI8	0.810
Boundary strength at home	BSH1	0.739
	BSH2	0.784
	BSH3	0.842
	BSH4	0.846
	BSH5	0.896
	BSH6	0.891
	BSH7	0.876
	BSH8	0.888
Need for recovery	NR1	0.775
	NR2	0.735
	NR3	0.772
	NR4	0.703
	NR5	0.716
	NR6	0.816
	NR7	0.866
	NR8	0.843
	NR9	0.869
	NR10	0.869
	NR11	0.856
Perceived organizational support	POS1	0.705
	POS2	0.724
	POS3	0.762
	POS4	0.773
	POS5	0.791
	POS6	0.818
	POS7	0.810
	POS8	0.803
	POS9	0.788
	POS10	0.762
Workplace vigor	WV1	0.772
	WV2	0.761
	WV3	0.676
	WV4	0.696
	WV5	0.753
	WV6	0.739
	WV7	0.801
	WV8	0.757
	WV9	0.708
	WV10	0.762
	WV11	0.719
	WV12	0.723
	WV13	0.777
	WV14	0.761

Notes: WRI = work-related irritation; NR = need for recovery; WV = workplace vigor; BSH = boundary strength at home; POS = perceived organizational support.

**Table A2:** Cross-loadings among latent constructs.

Items	WRI	BSH	NR	POS	WV
WRI1	**0.737**	−0.197	0.160	0.153	−0.134
WRI2	**0.689**	−0.175	0.200	0.184	−0.118
WRI3	**0.762**	−0.117	0.180	0.242	−0.132
WRI4	**0.679**	−0.154	0.153	0.117	−0.104
WRI5	**0.652**	−0.130	0.179	0.124	−0.049
WRI6	**0.760**	−0.105	0.199	0.169	−0.116
WRI7	**0.804**	−0.166	0.086	0.149	−0.070
WRI8	**0.747**	−0.121	0.207	0.152	−0.123
BSH1	−0.191	**0.723**	−0.078	−0.041	0.141
BSH2	−0.097	**0.695**	−0.270	−0.091	0.183
BSH3	−0.155	**0.759**	−0.257	−0.055	0.180
BSH4	−0.165	**0.779**	−0.207	−0.128	0.140
BSH5	−0.171	**0.844**	−0.168	−0.122	0.162
BSH6	−0.184	**0.827**	−0.191	−0.118	0.157
BSH7	−0.180	**0.824**	−0.182	−0.088	0.140
BSH8	−0.096	**0.824**	−0.201	−0.156	0.208
NR1	0.183	−0.185	**0.700**	0.125	−0.152
NR2	0.058	−0.158	**0.680**	0.133	−0.202
NR3	0.128	−0.079	**0.735**	0.182	−0.131
NR4	0.148	−0.087	**0.685**	0.079	−0.098
NR5	0.052	−0.099	**0.704**	0.130	−0.130
NR6	0.156	−0.145	**0.758**	0.160	−0.143
NR7	0.191	−0.279	**0.774**	0.123	−0.159
NR8	0.165	−0.229	**0.756**	0.099	−0.221
NR9	0.199	−0.158	**0.789**	0.192	−0.168
NR10	0.211	−0.190	**0.795**	0.160	−0.142
NR11	0.186	−0.148	**0.775**	0.195	−0.183
POS1	0.186	0.026	0.125	**0.703**	−0.030
POS2	0.096	0.025	0.180	**0.688**	−0.154
POS3	0.160	−0.061	0.122	**0.738**	−0.085
POS4	0.103	−0.163	0.136	**0.746**	−0.097
POS5	0.135	−0.039	0.130	**0.776**	−0.095
POS6	0.092	−0.101	0.114	**0.800**	−0.112
POS7	0.113	−0.098	0.157	**0.755**	−0.159
POS8	0.119	−0.083	0.155	**0.744**	−0.159
POS9	0.111	−0.145	0.119	**0.731**	−0.147
POS10	0.182	−0.166	0.099	**0.697**	−0.125
WV1	−0.049	0.089	−0.116	−0.157	**0.745**
WV2	−0.037	0.082	−0.116	−0.083	**0.749**
WV3	0.023	0.007	−0.138	−0.087	**0.679**
WV4	−0.068	0.122	−0.105	−0.092	**0.666**
WV5	−0.029	0.194	−0.062	−0.050	**0.744**
WV6	−0.088	0.148	−0.093	0.032	**0.738**
WV7	−0.082	0.111	−0.155	−0.032	**0.778**
WV8	−0.188	0.104	−0.115	−0.209	**0.684**
WV9	−0.138	0.085	−0.156	−0.131	**0.652**
WV10	−0.095	0.105	−0.116	−0.147	**0.723**
WV11	−0.038	0.141	−0.151	−0.115	**0.676**
WV12	−0.044	0.055	−0.121	−0.142	**0.702**
WV13	−0.125	0.123	−0.112	−0.075	**0.748**
WV14	−0.129	0.094	−0.122	−0.075	**0.734**

Notes: Values in bold represent the item’s primary loading (i.e., the highest factor loading across constructs). WRI = work-related irritation; NR = need for recovery; WV = workplace vigor; BSH = boundary strength at home; POS = perceived organizational support.
